# Using Electronic Patient Records to Discover Disease Correlations and Stratify Patient Cohorts

**DOI:** 10.1371/journal.pcbi.1002141

**Published:** 2011-08-25

**Authors:** Francisco S. Roque, Peter B. Jensen, Henriette Schmock, Marlene Dalgaard, Massimo Andreatta, Thomas Hansen, Karen Søeby, Søren Bredkjær, Anders Juul, Thomas Werge, Lars J. Jensen, Søren Brunak

**Affiliations:** 1 Center for Biological Sequence Analysis, Department of Systems Biology, Technical University of Denmark, Lyngby, Denmark; 2 NNF Center for Protein Research, University of Copenhagen, Copenhagen, Denmark; 3 Institute of Biological Psychiatry, Mental Health Center Sct. Hans, Copenhagen University Hospital, Roskilde, Denmark; 4 Department of Growth and Reproduction GR, Rigshospitalet, Copenhagen, Denmark; 5 Department of Clinical Biochemistry, Hvidovre Hospital, Copenhagen University Hospital, Hvidovre, Denmark; 6 Psychiatry Region Sealand, Ringsted, Denmark; Vanderbilt University, United States of America

## Abstract

Electronic patient records remain a rather unexplored, but potentially rich data source for discovering correlations between diseases. We describe a general approach for gathering phenotypic descriptions of patients from medical records in a systematic and non-cohort dependent manner. By extracting phenotype information from the free-text in such records we demonstrate that we can extend the information contained in the structured record data, and use it for producing fine-grained patient stratification and disease co-occurrence statistics. The approach uses a dictionary based on the International Classification of Disease ontology and is therefore in principle language independent. As a use case we show how records from a Danish psychiatric hospital lead to the identification of disease correlations, which subsequently can be mapped to systems biology frameworks.

## Author Summary

Text mining and information extraction can be seen as the challenge of converting information hidden in text into manageable data. We have used text mining to automatically extract clinically relevant terms from 5543 psychiatric patient records and map these to disease codes in the International Classification of Disease ontology (ICD10). Mined codes were supplemented by existing coded data. For each patient we constructed a phenotypic profile of associated ICD10 codes. This allowed us to cluster patients together based on the similarity of their profiles. The result is a patient stratification based on more complete profiles than the primary diagnosis, which is typically used. Similarly we investigated comorbidities by looking for pairs of disease codes cooccuring in patients more often than expected. Our high ranking pairs were manually curated by a medical doctor who flagged 93 candidates as interesting. For a number of these we were able to find genes/proteins known to be associated with the diseases using the OMIM database. The disease-associated proteins allowed us to construct protein networks suspected to be involved in each of the phenotypes. Shared proteins between two associated diseases might provide insight to the disease comorbidity.

## Introduction

With the consolidation of EPR systems in modern healthcare, massive amounts of clinical data and phenotype data are gradually becoming available for researchers [1], [2], [3], [4], [5], [6]. Alone, or integrated with existing biomedical resources, these EPR systems constitute a rich resource for many types of data driven knowledge discovery as we demonstrate in this paper. In the coming years, as these data are also coupled to the expected explosion in personal genomic data, the translational meeting of ‘bench and bedside’ is expected to push scientific advancements in personalized medicine [4], [7], [8], [9], [10].

EPR systems document patient morbidity, treatment and care over time. They comprise different types of structured and unstructured data, ranging from coded diagnoses, ordinary physiological measures, biobank data, laboratory test results over medication prescriptions, and treatment plans, to free text notes such as admission notes, discharge notes and nursing notes [11], [12].

We focus here on the assigned structured diagnosis codes and the free text notes. In our Danish setting, assigned codes are coded in the EPR according to the International Classification of Disease version 10 (ICD10), and are ultimately reported to the discharge registries for reimbursement. This process has known (but poorly quantified) biases since codes result in different reimbursement sums [13], [14]. Assigned codes will also typically pertain strictly to the current hospitalization and the morbidity deemed strictly relevant to it. These bias and completeness issues are also documented in insurance claims data with ICD9 [15]. In contrast free text notes should not have this bias, and contain much additional information, but in an inherently unstructured form (refs). In this paper we demonstrate how text- and data mining techniques can be used to extract clinical information hidden in text to augment coded data. The result is a much more complete phenotypic description of patients, than what could be obtained from just structured data and registries.

There is an increasing focus on the research potential of both structured and textual data collected in EPR systems and registries. Examples of this work is classical database knowledge discovery and association mining [16], [17], [18], identifying and classifying specific medical cases or conditions in an EPR [19], [20], [21], [22], patient safety and automated surveillance of adverse events, contraindications and epidemics [23], [24], [25], comorbidity and disease networks [26], [27], [28], autocoding of clinical text [29], [30], [31], [32], medication information extraction [33], [34] and identifying suitable individuals for clinical trials [35], [36]. Also see review by Meystre et, al [37]. Some of this work deals strictly with structured data, while some use text mining techniques to extract information from text. Much of the latter work builds on existing Natural Language Processing (NLP) text mining tools designed for recognizing clinical terms and findings and mapping them to controlled vocabularies such as the United Medical Language System (UMLS). Some of these tools are MedLee, MetaMap, cTakes and HITEx ([29], [38], [39], [40]). For Danish text, unfortunately no such EPR Information Extraction tools exist. To extract data from the text for our analysis, we therefore constructed our own text mining module compatible with Danish classification resources and easily adapted to any language with a translation of ICD10. Our comparatively simple approach significantly enriches structured EPR data, and allows a higher resolution analysis than otherwise possible.

Independently of the research assisted by the information presented in the patient records, several approaches have been developed to discover novel disease associations, either based on shared disease causing genes or on overlapping pathways [26], [41], [42]. Known disorder–gene associations from available resources like OMIM have been used to establish links between diseases, thus creating a network of disorders [26]. Common to many of these approaches is the extensive use of protein-protein interactions from large-scale proteomic studies. Linking disease-gene information with the growing data present in EPR systems will allow for a better understanding of disease etiology and phenotype-genotype associations. The PheWAS work at Vanderbuilt University. [43], [44] is a recent illustration of this.

Here we describe a strategy for exploring EPR data from a patient cohort in the context of subsequent systems biology analysis. By mining the free-text parts of the EPR from a psychiatric hospital we are able to augment the disease information assigned in structured formats as ICD10 codes, and thus obtain a much richer phenotype profile of each patient. Treating these profiles as phenotype vectors [41] in the controlled vocabulary space of the ICD10 disease classification, we demonstrate how they can be used to investigate disease comorbidity and patient stratification, paving the way for discovery of the underlying molecular level disease etiology in the form of overlapping genes and pathways. A longer-term perspective is to also include genetic profiles of the individuals in these data integration schemes, but this is not explored in the present paper.

## Results

### Validation of the text mining approach

We based our study on a corpus of 5,543 patient records from the Sct. Hans Hospital (the largest Danish psychiatric hospital) collected in the period 1998–2008. A manually curated subset of the records was used to assess the precision of the text mining approach. From structured fields in the EPR, we extracted 31,662 ICD10 codes, representing 351 different level 3 codes and corresponding to 2.7 unique codes associated to each patient on average. In the selected text found in the EPR our text mining approach matched 218,963 text strings to strings in a compiled dictionary of ICD10 terms and generated term variants (see Materials and Methods and Text S1 for additional detail). A further 22,956 matches were disqualified by a negation module whenever a negating word or mention of another subject (e.g. mother, sister or friend) was found in the preceding part of the sentence. The corresponding codes of these terms covered 554 different level 3 ICD10 codes, on average 9.5 unique codes per patient. Combining mined and assigned codes results in 674 different ICD10 codes with 12.3 average codes per patient The combined data was gathered in a Patient-ICD10 association matrix, by assigning each Patient–ICD10 combination both a binary and a TF-IDF ([45]) weighted value indicating whether or not a given code was associated with a given patient and how strongly. Rows thus represent the morbidity of a patient as a vector in ICD10 space, and columns represent the prevalence of a ICD10 as a vector in patient space.

The precision of our text mining was quantitatively assessed by manually checking all 2,724 mining hits for 48 patients (Table 1). The validation set covered 214 full level ICD10 codes, corresponding to 151 level 3 codes. A hit was considered correctly assigned when it was possible to infer a direct clinical link between the term and the patient from the record context. We defined precision in two ways: Incidence precision of all curated hits, and association precision, where an ICD10 code is considered correctly associated with a patient if it h77as at least one correct incidence. In both cases we considered how the precision was distributed among the different chapters. We found a total incidence precision of 87.78% and an association precision of 84.03%. False text mining hits fall in the categories: Negations, 3.9%; false subject, 0.6%; Delusion, 0.3%; Putative, 1.5%; Polysemic, 0.3%; Information to patient, 3.3%; Other, 2.2% (see Text S1). For the same 48 patients we also manually curated the 411 hits (373 negations and 38 subject) disqualified by the negation module. 330 of these were correctly disqualified giving an 80% precision of the negation module. 122 text mining hits out of 2,724 are due to hits categorized as negations or false subject that were not caught by the negation module. Combining the numbers the negation module identifies 73% of all relevant negations (330/(330+122)). The negation module is similar to the approach of the NegEX method [46], [47]. A further breakdown of the validation is available in Text S1.

**Table 1:** Precision of text-mining associations.

	Incidence precision (#mining hits)			Association precision (#ICD10 codes)		
Chapter	Correct	False	Precision	Correct	False	Precision
I	7	10	41.18%	7	6	53.85%
II	0	1	0.00%	0	1	0.00%
IV	30	4	88.24%	17	4	80.95%
V	486	20	96.05%	128	7	94.81%
VI	124	16	88.57%	46	9	83.64%
VII	19	13	59.38%	11	9	55.00%
IX	26	11	70.27%	13	5	72.22%
X	78	11	87.64%	36	4	90.00%
XI	67	12	84.81%	19	2	90.48%
XII	73	10	87.95%	29	9	76.32%
XIII	57	2	96.61%	17	2	89.47%
XIV	12	2	85.71%	6	1	85.71%
XVIII	1234	115	91.48%	252	53	82.62%
XIX	141	101	58.26%	36	8	81.82%
XX	4	0	100.00%	3	0	100.00%
XXI	33	5	86.84%	27	3	90.00%
All	2391	333	87.78%	647	123	84.03%

Precision is the number of true positives divided by the sum of true and false positives. Incidence precision distinguishes every individual mining hit as either correct or false. In association precision each ICD10 code is counted just once per patient and is considered correct if just one of the incidences of the code with this patient is correct. The final row contains the precision over all chapters.

### Comorbidity

**Figure 1:**
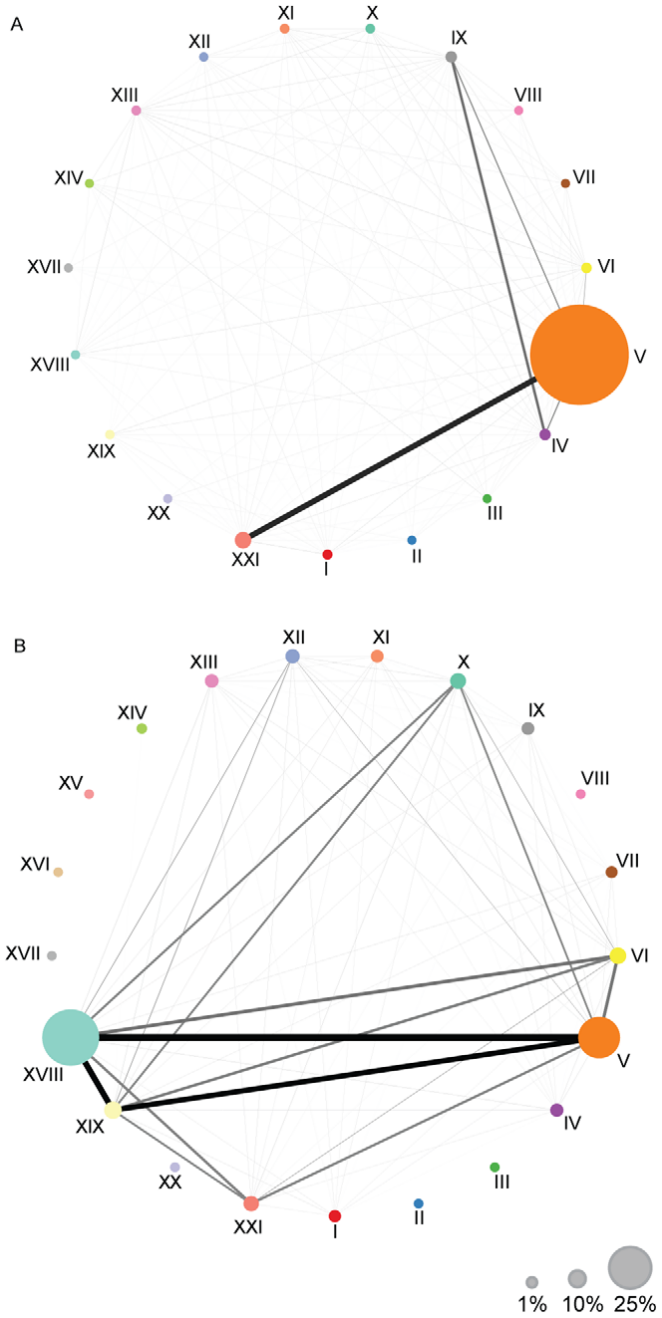
Disease chapter networks. ICD10 Chapters are shown as nodes; links represent correlations. Link weight represents correlation strength between two chapters; node area represents the proportion of codes from that chapter in the entire corpus. (A) Network based on the assigned codes for each patient. Most frequent chapter is chapter V ‘Mental and behavioral disorders’ with a frequency of 81%. The strongest correlation is between chapters V and XXI with a cosine similarity score of 0.45. Chapters IX, ‘Diseases of the circulatory system’ and IV ‘Endocrine, nutritional and metabolic diseases’ have a score of 0.3. (B) Full network containing both the assigned and mined codes for all patients. Chapters V and XVIII have a frequency of 24% and 35% respectively, and have a score of 0.92. After mining, ‘Diseases of the respiratory system’ - chapter X, and ‘Injury, poisoning and certain other consequences of external causes’ - chapter XIX, now have a cosine similarity score of 0.6 and 0.78, respectively.

ICD10 is organized into 22 chapters according to disease areas (see Materials and Methods). To discover the degree of comorbidity between chapters, we constructed an ICD10 chapter network (Figure 1). Based on which diseases belonging to a specific chapter each patient has in the corpus, we calculated a similarity score between the different chapters, ranging between 0 (for the lowest comorbidity), to 1 (highest comorbidity), see Materials and Methods. Codes for chapter V ‘Mental and behavioral disorders’ account for over 80% of the assigned codes given by physicians at Sct. Hans Hospital, while codes for chapter XXI ‘Factors influencing health status and contact with health services’ have a frequency of around 7%. These are also the two most correlated chapters. The strong correlation between mental disorders of chapter V and the observational Z-diagnoses of chapter XXI is most likely explained by a large ward in the hospital for forensic psychiatry, where patients are frequently admitted for mental observation following a criminal offence.

When including both the assigned and the mined codes from the textual records we capture many symptomatic descriptions for diseases. As seen on Figure 1b, more than 35% of all codes are pertaining to chapter XVIII ‘Symptoms, signs and abnormal clinical and laboratory findings, not elsewhere classified’, e.g. general medical complaints, edema, back pain, and elevated blood glucose. Chapter XIX ‘Injury, poisoning and certain other consequences of external causes’, as well as chapter XVIII, exhibit a high correlation with chapter V. Assigned codes are often restricted to the principal psychiatric illness and important for billing and social purposes, not necessarily reflecting the actual psychiatric treatment and care, nor the somatic disorders affecting the patient. For this reason, introducing the mined codes in the analysis allows capturing correlations that were previously impossible to find.

In our attempt to identify pairs of interesting unexpected co-morbidities, as well as general trends of correlation, we investigated pairs of ICD10 code vectors in patient space (columns in the patient-ICD10 association matrix). We used two measures to rank the 226,801 possible pairs of the 674 ICD10 codes, according to their co-association, compared to what would be randomly expected. Pairs were sorted based on p-values and a cut-off was imposed based on a comorbidity score and a false discovery rate of 1% (see Materials and Methods). The result is a list of 802 candidate ICD10 diagnostic pairs that occur more than twice as often as expected by random, and that are statistically significant at a false discovery rate of 1% (Data S1).

**Figure 2:**
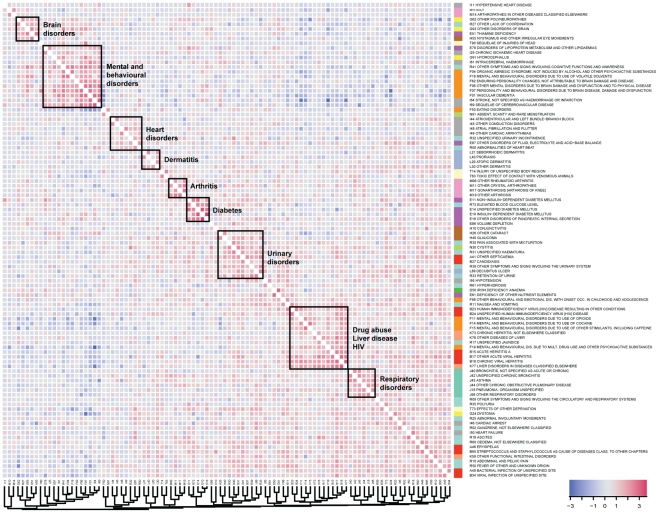
Disease-disease correlations. Heatmap of the most significant 100 ICD10 codes, based on ranking the list of 802 candidate pairs by their comorbidity scores. Chapter colors are highlighted next to the ICD10 codes. Diseases that occur often together have red color in the heatmap, while those with lower than expected co-occurrence are colored blue. The color label shows the log2 change of comorbidity between two diseases when compared to the expected level.

Using the comorbidity score as a similarity measure we clustered all 674 ICD10 codes and created a corresponding heatmap of the comorbidity scores for the ICD10 pairs. Figure 2 shows a truncated version of the entire heatmap, containing the scores of all the interactions for the top ranking 100 ICD10 codes (i.e., the top 100 codes found when sorting the list of 802 candidate pairs by their comorbidity score). The full heatmap for all 674 ICD10 codes extracted from the corpus can be inspected in Figure S1.

Figure 2 illustrates the general ability of our approach to capture correlations between different disorders. Several clusters of ICD10 codes relating to the same anatomical area or type of disorder can be identified along the diagonal of the heatmap. They range from trivial correlations (e.g., different arthritis disorders), to correlations of cause and effect codes (e.g., stroke and mental/behavioral disorders), to social and habitual correlations like drug abuse with liver diseases and HIV. Another interesting observation on the composition of the corpus is the lower than expected co-occurrence between the codes of the ‘mental and behavioral disorders’ cluster and the ‘drug abuse, liver disease, HIV’ cluster, as indicated by the blue areas in the upper and lower corners. These are very different groups of disorders that strongly stratify the patient corpus, and inspection of the specific diagnoses indicates that the correlation reflects two of the primary causes for admittance to the Sct. Hans Hospital (i.e., two distinct clinical departments): psychiatric disorders caused by stroke or brain injury, and mental illness accompanied by drug abuse.

Our approach will, and should, for the most part return trivial or already known co-morbidities. This is a result of the non-independence of ICD10 codes. These will to a certain extent be expected to correlate according to anatomical and functional similarity, which again is what the taxonomy of the ICD10 classification attempts to capture. This is also reflected in figure 1 where e.g. chapter V, Mental and behavioral disorders and chapter VI, Diseases of the nervous system exhibit correlation. One could attempt to reduce this type of dependency, by imposing filters for intra chapter pairs, or in other ways use the taxonomy as a filter or weighing scheme [48]. However since the candidate list resulting from the described pipeline was manageable for manual curation, we choose to not impose further filtering with the risk of losing interesting comorbidities. Trivial pairs occur for example between two codes for essentially the same disease (e.g., E11 ‘Non-insulin-dependent diabetes mellitus’ and R73 ‘Elevated blood glucose level’), between trivial disease-symptom pairs (e.g., N30 ‘Cystitis’ and R30 ‘Pain associated with micturition’), or between pairs of well-established correlations (e.g., E51 ‘Thiamine deficiency’ and H55 ‘Nystagmus and other irregular eye movements’). To discriminate potentially interesting, novel candidate co-morbidities from the many trivial ones, an experienced medical doctor manually inspected the candidate list of 802 pairs and flagged 93 surprising co-morbidities A list of all code pairs as well as flagged pairs can be seen in Supplementary Data S1.

Disease correlations may or may not have genetic causes. To identify a possible molecular basis for the flagged pairs, we extracted genes implicated in those particular diseases when a good mapping from ICD10 to OMIM was possible (see Materials and Methods). We then created a protein-protein interaction network by determining the first order interactions of those genes in refined experimental proteomics data (see Materials and Methods). For each disease pair, we searched for shared first order interactions connecting the two networks. Despite the difficulty of mapping the different terminologies and genes with this approach [27], the analysis revealed several connected proteins which are novel in relation to the diseases used to generate the networks. For example, we narrowed down an interesting case story between Alopecia (i.e., hair loss, ICD10 L65) and Migraine (ICD10 G43). We found that THRA, thyroid hormone receptor, not previously associated with any of the two diseases, is a shared interaction partner of Protein Hairless (HR, a putative single zinc finger transcription factor protein) involved in alopecia [49], and the Estrogen Receptor 1 (ESR1) associated with migraine [50], with a p-value of 1.17×10^−3^ (Materials and Methods). This may suggest that these two diseases share a similar molecular mechanism of action. A network view of these proteins and their interaction partners can be seen on Supplementary Figure S2. Migraine and alopecia were associated to 210 and 38 patients respectively, with 12 cooccurences (comorbidity score of 1.92, p-value of 2.07×10^−6^). To confirm these associations, which primarily came from text mining, we checked the surrounding textual contexts of all the mining associations to check their validity. For the 12 overlaps a medical doctors looked for confirmation in the full EPR record. In the case of migraine, in some cases a more correct clinical description would have been ‘headache’, and for alopecia some cases covered fear of or delusion of hair loss. The corrected contingency numbers were 168 (migraine), 26 (alopecia), 9 (both), and results in a comorbidity score of 0.4 and a p-value of 2.81×10^−6^. Of the remaining 9 patients with migraine and alopecia, six are women aged 21–63 and three are men aged between 47 and 54.

The observed comorbidity may reflect different side effects from medication [51], [52], [53]; most prominently seen with SSRIs (Selective Serotonin Reuptake Inhibitors for treatment of depression) that have been associated with cutaneous reactions, including alopecia, and migraine [54]. Also, frequently prescribed oral contraceptives are associated with migraines [55]. In fact, inspection of the nine comorbidity cases revealed that three patients were being treated with SSRIs (with a possible link to hair loss mentioned in the medical notes), two patients were administered oral contraceptives and one patient was treated with calcium antagonists and antiepileptic drugs. Removing 3 of the co-morbid cases corresponding to the SSRI treated patients results in a recalculated p-value of 2.9×10^−4^.

The comorbidity may also have an etiological cause that relates to schizophrenia, the primary disease of the patients. It has previously been shown that schizophrenia is associated with celiac disease, i.e. the highly under-diagnosed condition of gluten allergy [56], which in turn has been linked to both alopecia, and migraine; in fact the two latter conditions are now indications for diagnostic work-up for celiac disease according to the recent guidelines from the American Gastroenterological Association [57], [58].

### Patient stratification

In a specific hospital corpus the most important level of stratification is generally based on the primary diagnosis, or inclusion, which dictates treatment and care. The stratification can be very specific and based on lab results and tests for molecular markers, such as in the case of hormone receptor variants in breast cancer [59]. We were interested in determining if the combined mined and structured data could lead to a richer structure in the patient population, spanning a wider range of phenotypes, not typically considered when stratifying a specific corpus by assigned codes.

In the patient-ICD10 association matrix each patient is represented as a vector of associated ICD10 codes in the space of all the 674 ICD10 codes. We calculated cosine similarity [41] between the ICD10 vectors of all possible pairs of patients, and used this as the basis for a hierarchical clustering of patients. We used TF-IDF [45] weighted values in the association of each ICD10 code to the vector of a patient. (see Materials and Methods).

**Figure 3:**
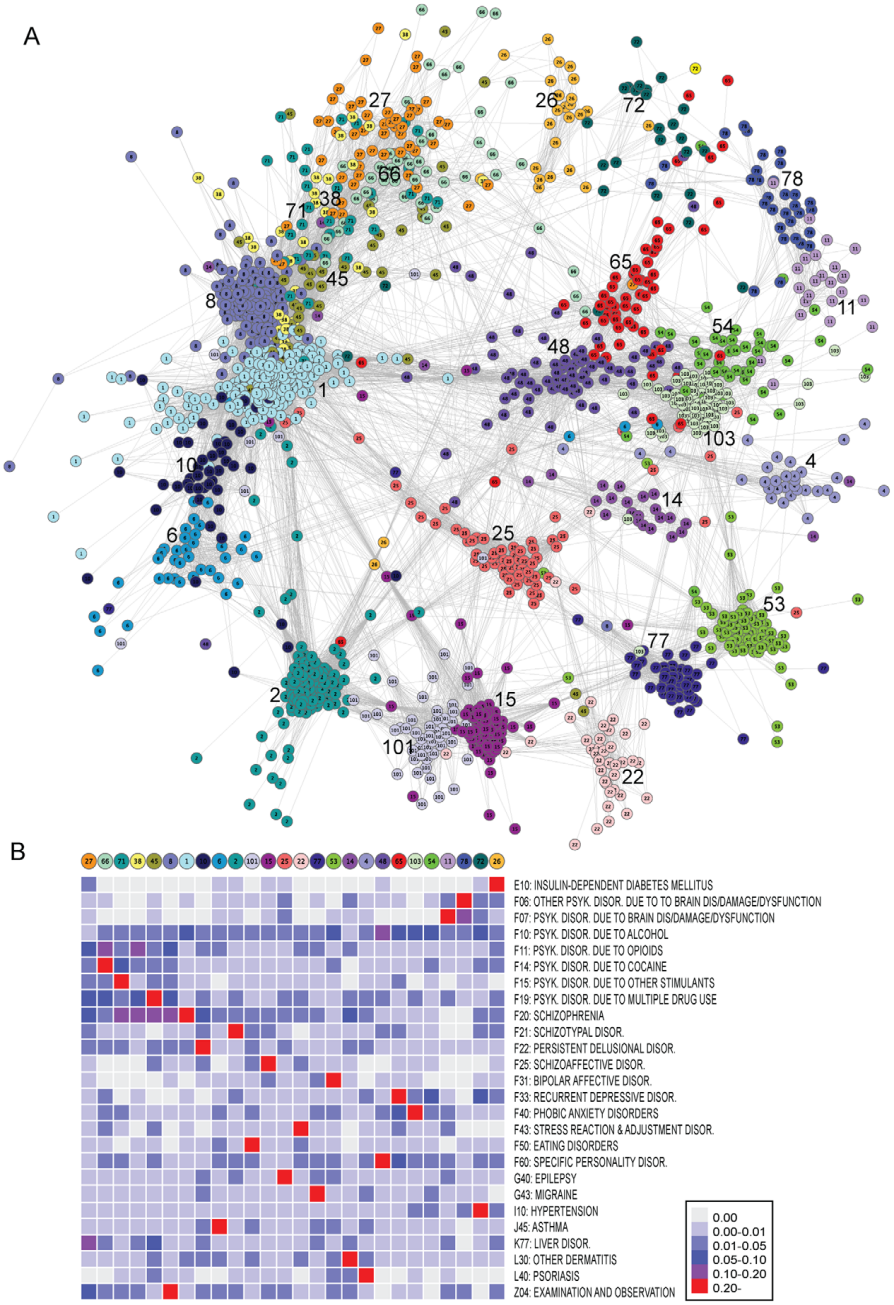
Patient cohort network. (A) Nodes represent 1,497 patients from 26 clusters. Edges are correlations between patients. Node color denotes cluster membership. (B) Heatmap showing ICD10 composition of each cluster. Values are the fraction of the cluster ICD10 vector covered by this code. Shown are only the 26 ICD10 codes that are most distinguishing codes for a cluster. The heatmap columns match the network clusters in a counter clockwise direction starting at cluster 27.

Figure 3 shows those 26 clusters with at least 25 members resulting from the clustering. They are laid out according to the patient-patient similarity and colored by group membership. The ICD10 characteristics of each group are seen in figure 3b (see Materials and Methods). In all but one cluster, 54, a single ICD10 code stands out as the most discriminating code. The TF-IDF value for this code constitutes up to 18–40% of the sum of all TF-IDF values in the vector. Furthermore, no two clusters share the same main code. The ICD10 characteristics of each cluster are shown in Figure 3b. From this figure, we see that Schizophrenia has a strong component in several clusters, primarily located in the top left of the network. As pictured, many of these clusters are also characterized by various codes for alcohol/drug use, indicating the type of abuse as a good sub-stratification of schizophrenia. Similarly, alcohol seems to be a common denominator for clusters 48–54, which are primarily characterized by depressive disorders, anxiety disorders, and other personality disorders. What is also interesting is that many patients fall into clusters characterized by somatic codes like diabetes and psoriasis, which have certainly not been the initial reason for admittance to the hospital. This is largely attributable to data coming from text mining (see Supplementary Data S1).

## Discussion

As EPR systems become the norm in modern health care, focus is naturally turned to exploring this treasure trove of data for improving health care and research [60]. Extracting the data is a first step, and as EPR systems in many countries maintain the use of free text to complement structured data, text-mining approaches are necessary for extracting data usable in further analyses.

The enrichment of existing structured patient data by text mining significantly expands phenotype profiles, both within the specific pathology of the corpus, but especially into other disease areas. We present one example of comorbidity between two diseases that are very often not coded in the record by the physician, but show up in the patient record text and are later picked up by mining. The enrichment from mining is also visible in our attempts to stratify patients, where potential is shown for uncovering additional layers of the population structure. More detailed stratification of patient cohorts could help improve population homogeneity and signal strength in genome wide association studies, and lead to increased power in case-control studies [35], [36].

The procedure described here represents, in our opinion, a practical non-hypothesis driven approach for extracting valuable information from patient records for any patient corpus where manual inspection and ICD10 association would turn into an otherwise impossible task. Furthermore, we show how this information can be used in researching disease comorbidity and patient stratification and how it can be mapped to the underlying systems biology revealing possible causes for the observed correlations.

The results obtained from a data driven approach like this one will obviously depend on the composition and domain of the patient corpus and on the amount and quality of the available data. In that sense, some of the found correlations and results will be domain or cohort specific, and do not necessarily translate to general population wide conclusions. In the case of patient stratification, this is inherently true. Even in these cases however, novel correlations can still be highly valuable and suggest hypothesis for causality within the cohort in terms of treatments, procedures, responses, and co-morbidities that are not necessarily genetically founded.

## Materials and Methods

### Ethics statement

Patient data was analyzed anonymously and the project was ethically approved by the Danish National Board of Health (No. J. nr. 7-604-04-2/33/EHE).

### Patient corpus

The patient population data was collected from the Sct. Hans Mental Health Centre, in Roskilde, Denmark. All analyses were performed on an anonymous data set. A total of 5,543 patients were followed from 1998–2008, and their records stored in an EPR database. 70% of the patients (4,822) are from the Copenhagen area, 61% of these are males. The average age is 30 years. The records are a mixture of structured diagnose assignments of ICD10 codes, ATC codes (http://www.whocc.no/atc) for medication usage, patient care notes from nurses and doctors, admission and personal information, etc. A corpus was created from the relevant tables of the Sct. Hans EPR, containing all unique text entries for each patient that were verified and signed by a physician. To each entry we assign an entry date, the note type, and the text. The note type identifies the type of text entry, such as the epicrisis, discharge note, treatment note, nursing note etc. A few non-medical notetypes such as ‘Social worker’ notes were excluded. In total, the corpus contains text for 4,765 patients with an average of 25,000 words per patient. In addition, we extracted all ICD10 codes assigned to patients that were stored in a structured format.

### ICD10 dictionary

The dictionary used in our text mining approach is based on the Danish translation of the Danish translation of the WHO International Classification of Diseases (ICD10), downloaded from the Danish National Board of Health the 2^nd^ Nov 2009. The ICD10 classification is a hierarchical classification of diseases and symptoms, divided into 22 anatomical/functional chapters with increased specification of terms in each lower level. The Danish translation of ICD10 consists of 22,261 terms, each uniquely matched to a code of between 3–5 characters. To increase the scope of the dictionary, we augmented existing terms with variants created by simple rules reflecting common semantic structures ([61], [62]) in the Danish ICD10 terms. E.g. adding truncated versions of terms containing specifiers like ‘.. forårsaget af ..’ (caused by), keeping just the preceding part. Terms containing commas and parenthesis are treated similarly. These variant terms are mapped to the same code as their parent. Since truncation throws away the detailed information in the case of low-level code-term pairs we ensure the code-term information content by rounding all codes to level 3. In this way all terms are essentially treated as synonyms of the more generic level 3 meaning. With variants the final dictionary consisted of 53,452 terms. Generated term variants were responsible for 24% of the total number of hits. More detail about the ICD10 dictionary is available in 1.

### Text mining

For relevant reviews on methods in text mining see e.g. ([37], [63], [64], [65]). The compiled text for each patient was normalized for orthographic variation like the dictionary, and a simple sentence splitter was used to split the text into smaller units. For each unit, a stepping algorithm created all possible strings of 1–10 words and looked them up in the dictionary. Exact matches were required. The longest possible match was always chosen. Candidates matching a blacklist of polysemic or otherwise mis-informative terms were disqualified. Negations and false subject-term associations were handled by disqualifying matches when the preceding sentence contained tokens from a list of negations (‘never’, ‘no’, etc) and subjects (‘mother’, ‘friend’, etc). Validated performance characteristics were covered in the results section. Further details about the text mining approach and its validation is contained in Text S1.

### Chapter networks

For each disease we created a vector mapping its presence or absence from a patient record. This resulted in 22 vectors for each disease chapter. The pair-wise overlap between vectors was quantified by calculating the cosine of the angle between normalized vector pairs [41]. The result is a score between 0 and 1, mapping the comorbidity value of each of the chapter pairs. We also calculated the frequency of each chapter in relation to the total number of chapter assignments. In Figure 1, the roman numerals represent the different ICD10 chapter numbers: I, Certain infectious and parasitic diseases; II, Neoplasms; III, Diseases of the blood and blood-forming organs and certain disorders involving the immune mechanism; IV, Endocrine, nutritional and metabolic diseases; V, Mental and behavioral disorders; VI, Diseases of the nervous system; VII, Diseases of the eye and adnexa; VIII, Diseases of the ear and mastoid process; IX, Diseases of the circulatory system; X, Diseases of the respiratory system; XI, Diseases of the digestive system; XII, Diseases of the skin and subcutaneous tissue; XIII, Diseases of the musculoskeletal system and connective tissue; XIV, Diseases of the genitourinary system; XV, Pregnancy, childbirth and the puerperium; XVI, Certain conditions originating in the perinatal period; XVII, Congenital malformations, deformations and chromosomal abnormalities; XVIII, Symptoms, signs and abnormal clinical and laboratory findings, not elsewhere classified; XIX, Injury, poisoning and certain other consequences of external causes; XX, External causes of morbidity and mortality; XXI, Factors influencing health status and contact with health services; XXII, Codes for special purposes.

### Comorbidity ranking

For the purpose of exploring comorbidity between ICD10 codes we used two measures to rank the 226,801 possible ((674*674-674)/2) pairs of different codes, according to how often they come together in patients, compared to what would be randomly expected assuming no a-priori correlations. The two measures represent our desire to ensure statistical significance, while focusing on pairs with a noticeably increased co-association.

First, for each pair of ICD10 codes A and B, the patient corpus is divided and counted in the four categories: A & B, A NOT B, B NOT A and NOT A NOT B, according to their association to A and B. Using this, p-values are calculated using Fishers exact test, and the pairs are sorted accordingly. We then filtered this list by imposing a cut-off value of 1.0 of a comorbidity score between diseases A and B defined as:
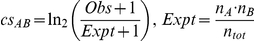
Where Obs is the observed number of ICD10 co-associations, and Expt is the expected number. Expected overlaps are calculated based on the prevalence of each disease in the actual corpus (n_A_ and n_B_). To make the tendency to favor pairs of low prevalence ICD10 codes less pronounced, a pseudo-count of 1 is added to nominator and denominator. Since we take log2 of this ratio, a cut-off value of 1.0 means we restrict our focus to pairs with a higher than two fold (approximately) over co-association. This comorbidity measure is very similar to the one used by Hidalgo et al. [66].

Finally we used a Benjamini-Hockberg false discovery rate method [67] on the ranked list to correct for multiple testing. The p-values for all pairs are multiplied by the total number of pairs (226,801) and divided by the rank of the pair in the sorted list. A cut-off is then imposed where the corrected p-value drops below 0.01. The result is a selection of 802 potentially interesting candidate pairs, with a false discovery rate of 1 percent, from the total of 226,801 pairs.

### Creating gene lists from ICD10 codes

There is no direct mapping between ICD10 codes and the OMIM [68] record entries. Furthermore, the disease names used by ICD10 and OMIM are not identical, so there was a need to map OMIM disease names into ICD10 codes. Work has been done mapping the online database and ICD9 codes, a previous version of the ICD [27]. We used the ICD10 to ICD9 General Equivalence Mapping available online from CMS (http://www.cms.gov/ICD10/) to map the ICD codes to their previous version. With the mappings in place, OMIM was parsed for phenotypic descriptions of defects in genes, as described in Lage et al., 2007 [41]. From the OMIM records, the *clinical synopsis* field was extracted for retrieving phenotypic descriptions regarding a certain disease. Additional information was retrieved from the *morbid map* tables, a map of disorders included in OMIM that have the syndrome name, chromosomal localization, and name of the disease causing gene. A manual curation step by a medical doctor ensured that each ICD10 code to be included in the analysis was assigned the correct OMIM entries.

### Genetic overlaps between ICD10 pairs

For each disease, a network was generated by taking the disease causing genes extracted from OMIM and determining their first order interactions in a human protein interaction network of refined experimental proteomics data. This procedure is described in detail elsewhere [41], [69], [70]. For determining genetic overlaps between two ICD10 diseases, we take their networks and identify those genes which are shared and have first order interactions with the seed genes. After a round of automatic overlap detection, we manually curated the results of the different steps in the pipeline, in order to detect erroneous assignments of disease names or genes, and reran the overlap detection in those cases. For those pairs where overlapping protein-protein interaction networks indicate underlying biological evidence, a final round of validation was done by manually checking if the binary associations from text mining of patients to the ICD10 codes were correct. Based on the corrected data, new p-values were calculated by Fishers exact test, and it was controlled that the p-value remained lower than the lowest p-value of the list of 802 candidates. The candidate genes found to overlap in the two disease networks were scored using the enrichment of OMIM seed genes in their first order interaction network, in a similar procedure as the one used by Lage et al., 2010 [69]. The score assigned to a candidate was the hyper geometric p value of observing the amount of interactions to the OMIM set out of all the interaction partners of the candidate. Our example of THRA has a total of seventeen interaction partners in the network, and two are with the input genes (HR and ESR1), having a p-value of 1.17×10^−3^.

### Patient stratification

By looking at the Patient-ICD10 matrix by rows, or patient vectors in ICD10 space, we can stratify patients based on the similarity of their ICD10 associations. Instead of a binary association of a given code to a given patient, we weighted the significance of ICD10 occurrences using the term frequency – inverse document frequency measure (TF-IDF) [45]. TF-IDF rewards high code frequency in the individual record, and penalizes high prevalence across the corpus. As a patient-patient stratification measure, we used the cosine similarity CS [41] to calculate the cosine of the angle between all pairs of vectors. We included only patients with at least three associated codes, and exclude a number of trivial/symptom codes (e.g., pain, coughing, itching). A total of 2,584 patients were found to have at least three associated codes. We used 1-CS as a distance measure and calculated average linkage clustering to divide patients into clusters. Manual inspection of the clustering dendrogram led us to cut the tree at a CS value of 0.6, which created a total of 307 clusters. 26 clusters contained 25 or more members, accounting for a total of 1,800 patients. Taking all edges with CS greater than 0.6 between these patients, the network in Figure 3a of 1,497 patients was created. The network layout is based purely on an edge weighted layout algorithm. In order to investigate the clinical characteristics of each cluster, we concatenated the assigned and mined data for all members of a cluster, and calculated a new TF-IDF code vector for the entire cluster in ICD10 space. Figure 3b illustrates these characteristics.

## Supporting Information

Figure S1 **All disease-disease correlations.** Heatmap of all 674 level 3 ICD10 codes found in the corpus. Chapter colors are highlighted next to the ICD10 codes. Diseases that occur often together have red color in the heatmap, while those with lower than expected co-occurrence are colored blue. The color label shows the log2 change of comorbidity between two diseases when compared to the expected level.
(PDF)

Figure S2 **Protein interaction network.** The putative single zinc finger transcription factor protein HR involved in alopecia and the Estrogen Receptor (ESR1) have thyroid hormone receptor (THRA) as a shared interaction partner.
(EPS)

Dataset S1 **Comorbidity candidate lists.** The complete list of 802 candidate comorbidity pairs resulting from sorting disease pairs on p-value, truncating based on comorbidity score (ln2(ratio)) and imposing a Benjamini Hochberg false discovery rate (FDR) of 1%. Also the list containing the 93 surprising co-morbidities flagged in manual curation by a medical doctor. Finally a table showing how the members of the 26 clusters in figure 3 are associated with the ICD10 code that is most distinguishing for that cluster. Mined contains those patients where the association comes only from mining, and assigned contains those patients where association comes from assignment only or from both assignment and mining. Cluster 54 contains 13 patients that are in fact not associated to F10 at all.
(XLS)

Text S1 **Supplementary text.** Detailed information about ICD10 dictionary generation and the text mining procedure and validation. Also additional information about genetic overlaps between ICD10 pairs.
(DOC)
