# Supplementary figures and images for: Using Electronic Patient Records to Discover Disease Correlations and Stratify Patient Cohorts
Source: PLoS Comput Biol. 2011 Aug 25;7(8):e1002141. doi: 10.1371/journal.pcbi.1002141 (PMC3161904; doi:10.1371/journal.pcbi.1002141)

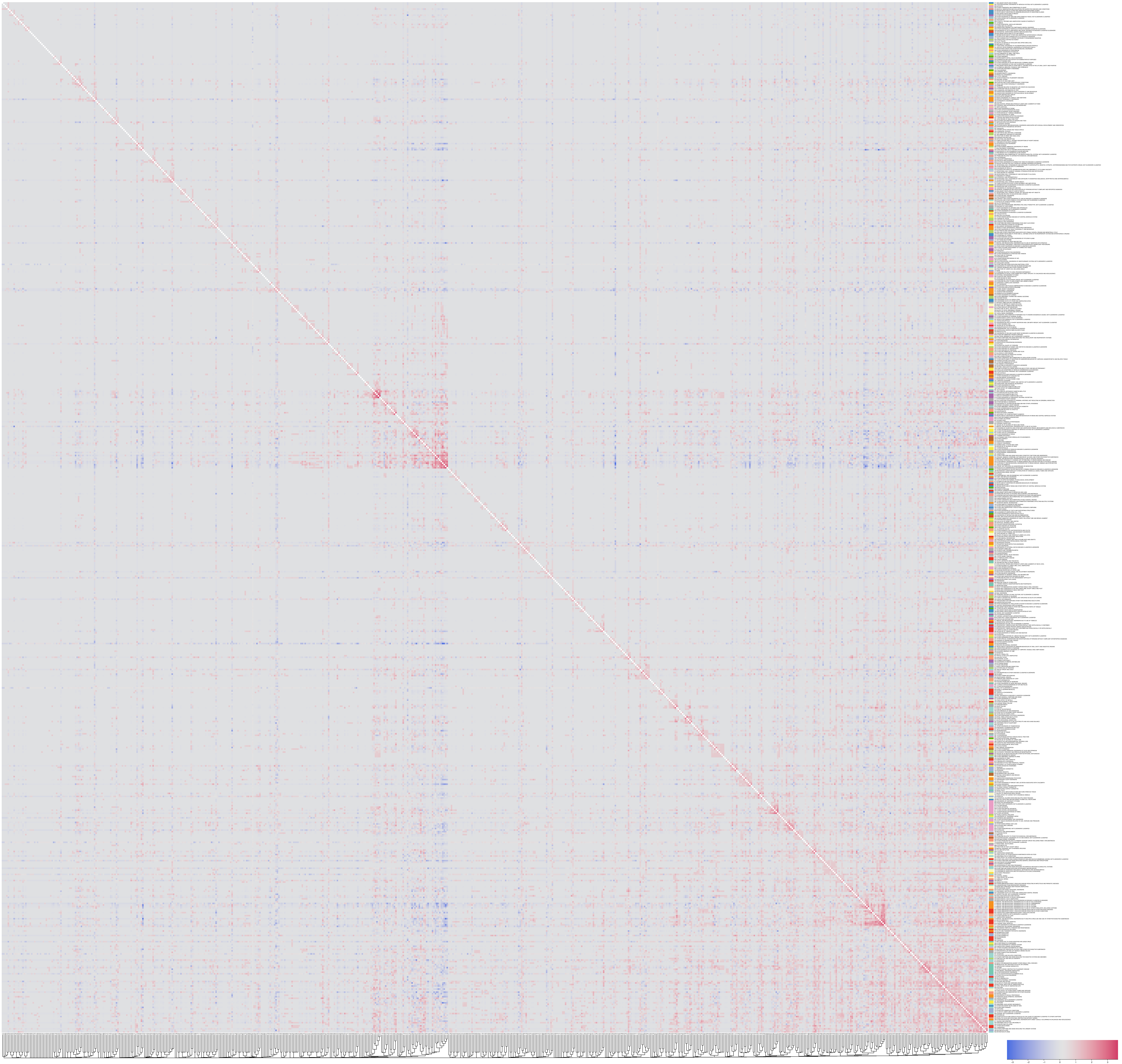

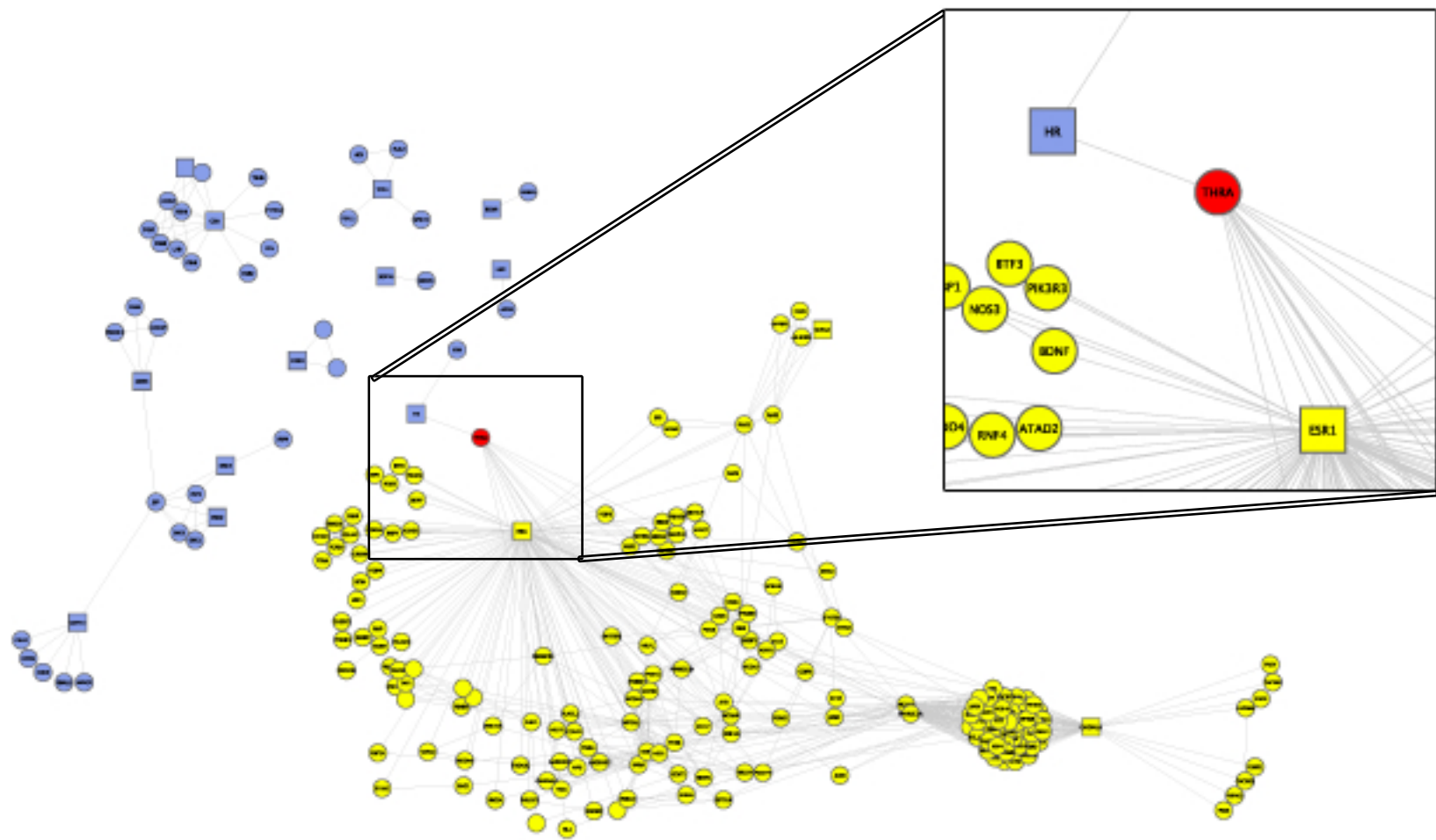

Supplement: Figure S1 — All disease-disease correlations. Heatmap of all 674 level 3 ICD10 codes found in the corpus. Chapter colors are highlighted next to the ICD10 codes. Diseases that occur often together have red color in the heatmap, while those with lower than expected co-occurrence are colored blue. The color label shows the log2 change of comorbidity between two diseases when compared to the expected level. (PDF) [file pcbi.1002141.s001.pdf]
