# Supplementary material for: Using Electronic Patient Records to Discover Disease Correlations and Stratify Patient Cohorts
Source: PLoS Comput Biol. 2011 Aug 25;7(8):e1002141. doi: 10.1371/journal.pcbi.1002141 (PMC3161904; doi:10.1371/journal.pcbi.1002141)
Supplement: Text S1 — Supplementary text. Detailed information about ICD10 dictionary generation and the text mining procedure and validation. Also additional information about genetic overlaps between ICD10 pairs. (DOC) [file pcbi.1002141.s004.doc]

**Supporting Text**

**Using electronic patient records to discover disease correlations and stratify patient cohorts**

Francisco S Roque^1†^, Peter B Jensen^2†^, Henriette Schmock^3^, Marlene Dalgaard^4^, Massimo Andreatta^1^ Thomas Hansen^3^, Karen Søeby^5^, Søren Bredkjær^3,6^, Anders Juul^4^, Thomas Werge^3^, Lars J Jensen^2^, and Søren Brunak^1,2*^

^1^Center for Biological Sequence Analysis, Department of Systems Biology, Technical University of Denmark, Lyngby, Denmark.

^2^ NNF Center for Protein Research, University of Copenhagen, Copenhagen, Denmark

^3^ Institute of Biological Psychiatry, Mental Health Center Sct. Hans, Copenhagen University Hospital, Roskilde, Denmark

^4^ Department of Growth and Reproduction GR, Rigshospitalet, Copenhagen, Denmark

^5^ Department of Clinical Biochemistry, Hvidovre Hospital, Copenhagen University Hospital, Denmark

^6^ Psychiatry Region Sealand, Ringsted, Denmark

^*^ To whom correspondence should be addressed (brunak@cbs.dtu.dk).

^†^ These authors contributed equally to this work.

**Dictionary generation**

The dictionary used in this study is based on the Danish translation of the WHO International Classification of Diseases (ICD10), downloaded from the Danish national board of health 2. Nov 2009. ICD10 is divided into 22 chapters, and has a hierarchical structure with increased specification of terms in each lower level. Each term is uniquely matched to a code of between 3 and 5 characters.

The core of the dictionary consists of all ICD10 terms in their original form in UTF8 format and uppercased. This is a total of 22,261 unique terms matched 1:1 with an ICD10 code. In addition to this, a number of permutations of the core terms are created, with each created term variant pointing to the same ICD10 code as the term it was derived from. These permutations are:

1. Comma permutation. Many terms contain a comma, and in a number of these the term structure is such, that the term maintains its clinical meaning by swapping the right and left side of the comma, or by keeping only what is on the left side. Example: A060 AMØBEDYSENTERI, AKUT -> A060 AKUT AMØBEDYSENTERI and A060 AMØBEDYSENTERI
2. Abbreviations. Terms containing a number of standard abbreviations are added in a version where the abbreviated word is written in full. Example: B029 HERPES ZOSTER U KOMPLIKATION -> B029 HERPES ZOSTER UDEN KOMPLIKATION
3. Parenthesis. Some terms contain a parenthesis, which typically contain some further specification of the term. A variant with the parenthesis deleted is added to the dictionary. Example: A00 KOLERA FORÅRSAGET AF VIBRIO CHOLERAE (KLASSISK KOLERA) -> A00 KOLERA FORÅRSAGET AF VIBRIO CHOLERAE
4. Typical expressions. Seven typical expressions used as specifiers, indicators of causative agents or to express lack of where identified in many terms. Again for our purposes this is typically not relevant, so a variant of these terms, with the expression and whatever follows, removed, is added to the dictionary. Example: B059 MÆSLINGER UDEN SPECIFIKATION -> B059 MÆSLINGER
5. ICD10 codes as terms. The ICD10 codes themselves are added as terms for their own code.

Finally all special characters are removed from the terms. Variants are created in an iterative way, in the order indicated above, such that type 3 permutations are also performed on the variations already created by type 1 and type 2 permutations. Some of the permutations result in addition of nonsense terms to the dictionary, but the very fact that they are nonsense terms makes them harmless. A more serious problem is the creation of variants that are sensible terms, but have lost the actual clinical meaning of the original term. Example: F019 DEMENS, VASKULÆR UDEN SPECIFIKATION -> F019 VASKULÆR UDEN SPECIFIKATION DEMENS -> F019 VASKULÆR. The first permutation is a comma permutation that leads to a nonsense term, which in the next permutation produces a term with single word vaskulær (vascular) pointing to F019. This is a simple word with no diagnostic or symptomatic meaning by itself, and can definitely not be said to be a synonym of F019. This type of variant is typically very short, so all dictionary terms of one and two words have been manually curated for this type of terms. They are not actually deleted from the dictionary but are added to a blacklist.

The objective of the dictionary construction is to get as many (sensible) terms as possible for each ICD10 code. Essentially they are synonyms of the original term. So the goal is to change the 1:1 relationship of ICD10 code to Term into a 1:many relationship. The permutation procedure inevitably results in situations where identical terms are created, terms that point to different ICD10 codes. In order to fix this, the complete dictionary is processed back into a 1:many structure by enforcing the following rules when two or more instances of the same term point to different ICD10 codes:

1. If the term is an original part of ICD10, then only the version pointing to the original ICD10 code is kept in the dictionary. Example:

DA37 KIGHOSTE

DA370 KIGHOSTE FORÅRSAGET AF BORDETELLA PERTUSSIS -> DA370 KIGHOSTE

Thus two codes with the same term are created, but since DA37 KIGHOSTE is an original, DA370 KIGHOSTE is deleted.

1. If the identical terms are all variations resulting from permutations, then it is checked if the ICD10 codes become identical at a higher level in the hierarchy, going to a maximum of level 3. If this is the case, one copy of the term is kept, and set to point to this ICD10 code. These contributions to the dictionary are counted as mixed types. If no common ICD10 stem is found, all the variations are removed. Example:

A395 HJERTESYGDOM FORÅRSAGET AF MENINGOKOKKER -> A395 HJERTESYGDOM

I518 HJERTESYGDOM ANDRE DÅRLIGT DEFINEREDE -> I518 HJERTESYGDOM

I519 HJERTESYGDOM UDEN SPECIFIKATION -> I519 HJERTESYGDOM

Permutations create 3 (actually more) identical terms HJERTESYGDOM pointing to 3 different codes. Ignoring the first term, the last two share a 3-character stem A51 that would have resulted in I51 HJERTESYGDOM to be added to the dictionary. However, since this stem is not shared with the top candidate A395, which points too a whole different chapter, the term HJERTESYGDOM is not added to the dictionary at all.

1. (The original ICD10 classification does in fact contain a few cases of the same term pointing to two different codes. In these instances, only the term pointing to the lowest level in the classification is kept.)

As a final addition to the dictionary, a number of terms have been added manually. These are obvious variants of frequent diseases missing from the dictionary that have been discovered during the process of working with the data, or variations with clinical sense that point to a wrong ICD10 code. This accounts for approximately 50 dictionary entries. The final dictionary consists of 53,452 terms.

**Compiling the text corpus**

Our copy of the Sct. Hans EPR system contains information on 5543 unique patients that have been in contact with the hospital in the period 1998-2008. Of these patients text notes exist for a total of 4765. The bulk of the remaining patients have only had passing association with Sct. Hans through e.g. a single blood sample being drawn. We extracted a total of 3082570 notes from the relevant database tables and for relevant notetypes. Notetypes included were:

Outpatient notes, treatment note, epicrisis, ergo/physiotherapy note, preexamination notes, group therapy notes, admission notes, psychological examination notes, psychotherapy notes, nursing notes, interdisciplinary notes, discharge note/summary, treatment plan and phone consultation. Only unique notes (from same date) were included and only notes signed by a physician. The combined notes for a given patients average approx. 25,000 words. For each note we had the possibility to enrich it with information of date and notetype. For each patient we stored all obtained text in a single line with tabs delimiting individual notes. Within each note we did a further segmentation of the text by applying a simple sentence splitter. Using regular expression sentence boundaries were identified by looking for periods and capitalization while checking against a large dictionary of abbreviations used in the corpus, that the boundary is not simply an abbreviation. Finally the text was normalized for orthographic variation by removing special characters and similar.

**Text mining ICD10 codes from the corpus**

The text corpus was parsed in units of sentences. The sentence is tokenized treating spaces as word boundaries. A stepping algorithm creates all possible candidate strings by concatenating from one to 10 adjacent tokens and looks each candidate up in the dictionary. A hit consists of a 1:1 match between candidate strings in the corpus and the complete set of dictionary terms. When multiple matching candidates are possible, the longest is always chosen. E.g. the sentence ‘he has paronoid skizofrenia’ will match both skizofrenia and paranoid skizofrenia so the longest is picked.

Candidates that match a dictionary term, and do not match any blacklisted terms (polysemic or mis-informative terms), are then checked for negations or family mention in the preceding tokens of the current sentence. We consider the negations ‘aldrig’, ‘ingen’, ‘intet’ , ‘uden’ (‘Never’, ‘None’, ’No’, ’Without’) and disregard candidates with any of these within the preceding sentence. We similarly look for any mention of another subject (‘son’, ‘father’, ‘friend’..) in the preceding sentence. This will also disqualify a candidate term, since very often the hit will pertain to that other subject and not the patient.

**The extracted data**

Using the text mining approach, we obtain hits covering a range of scenarios ranging from very specific phenotypes like ‘PARANOID SKIZOFRENI’ and ‘SOCIAL FOBI’ (paranoid schizophrenia and social fobia), to more general ones such as ‘HOVEDPINE’ (headache).

The majority of the hits fall in the general category. This category is generally characterized by terms consisting of only one word, typically describing a disease or symptom in its most generic form. Since created term variants point to the same ICD10 code as the term it is derived from, the permutation process will create some terms that point to an ICD10 code with an original meaning that is more specific than the variant term can support. That means a very specific code can be assigned to a hit with only a generic description of disease or symptom.

To deal with this, while also creating a more homogenous data material, all mined codes were converted to level 3 in the ICD10 classification. All subterms are treated as synonyms for the upper term. In some cases a very specific code could have been mined from a correct context, thus resulting in lost information, but in the more frequent case of a generic context, it increases the precision of the mined term. Furthermore, level 3 also represents a good general distinction of different diseases.

We text mined a total of 218,963 hits, where a candidate in the corpus matched a dictionary entry, and was not disqualified by the blacklist, negations or family mention. A total of 22,956 potential hits were disqualified by the negation module. That is close to 10 % of the total number of potential hits, and underlines the importance of accounting for this.

The hits cover a total of 1.229 different dictionary terms pointing to 1,064 different full level ICD10 codes. Rounding up to level 3 reduced the number ICD10 codes from 1,064 to 554. Out of the 4,765 patients in the Skt. Hans database where notes existed, hits were mined for 3.259 patients. The remaining patients either had no text entries, or the text entries were too few and/or short to generate any hits.

In addition to the mined ICD10 codes, assigned codes were also extracted from structured fields in the EPR system. 31,662 assigned codes were found for 2,803 patients representing 351 different ICD10 codes at level 3. Adding assigned and mined data, we find ICD10 codes for a total of 3,290 patients. Of these, 66 patients have contributions only from assigned codes, 487 have contributions only from mining, and the remaining 2,737 have contributions from both. In total 674 different ICD10 codes at level 3 are represented in the combined data. Counting each code once per patient and distinguishing between assigned and mined codes; we found the following number of unique code-patient associations (averages are all based on 3,290 patients):

|  | Number of ICD10 codes retrieved | Average retrieved codes per patient |
| --- | --- | --- |
| Assigned only: | 4974 | 1.51 |
| Mined only: | 31662 | 9.62 |
| Assigned-mined overlap | 3798 | 1.15 |
| Total | 40434 | 12.28 |

The percentage of assigned codes that are also recovered by mining the text is 43%.

For each patient a vector is created with a unique list of all codes, assigned or mined, associated with this patient. As seen from the table, mining adds almost 10 additional terms to the 2.6 terms coming from assigned codes.

**Validation of textmining**

The precision of our text mining was investigated by manually checking all 2724 mining hits for 48 patients. The validation set covered 214 full level ICD10 codes, corresponding to 151 level 3 codes. A hit was considered a correctly mined association when it was possible from the immediate record context to see directly (or deduce with good certainty) a clinical link between the term and the patient. We defined precision in two ways: Incidence precision of all curated hits, and association precision where an ICD10 code is considered correctly associated with a patient if it has at least one correct incidence. In both cases we also considered how precision was distributed among the different chapters as seen in table 1 in the paper. The low precision in chapter 1 (Infectious and parasitic diseases) is largely caused by a number of false associations of the term ‘AIDS’, which mostly comes from somatic delusions. In chapter 19 (Injury, poisoning and certain other consequences of external causes) which includes the term ‘bivirkninger’ (sideeffects), a standard expression stating that the patient has been informed of possible sideeffects, cause many false associations, resulting in the low precision. We found the total incidence precision to be 87.78% and the association precision to be 84.03%. False textmining hits fall in the categories: Negations, 3.9%; False subject, 0.6%; Delusion, 0.3%; Putative, 1.5%; Polysemic, 0.3; Patient information, 3.3%; Other, 2.2%

Negations cover negation constructs not caught by our negation detection. Eg. ‘Investigated for dyslexia, but nothing was found’.

Wrong individual covers cases where the association is really to another person. Eg. ‘An aquaintance of his recently died from a heartattact’

Delusions cover cases where patients are delusional about a disorder. Eg. ‘ pt. is paranoid about contacting AIDS’

Putative covers cases where the clinial link is vague, speculative or avaiting confirmation. Eg. ‘pt should be examined by an ophthalmologist on suspicion of glaucoma’. Polysemic covers cases where a term is used in a nonclinical context. In the sample the ambiguity came from a geographical location that is also a clinical term.

Patient information covers information delivered to patient where the clinical association is eg. a future issue. Eg. ‘pt. has been informed of the possible sideeffects of the new drug’. Other covers any other false association.

**Validation of negation module**

For the same 48 patients we also assessed the precision of the negation module by manually curating the 411 text mining hits that were disqualified. 373 of these were due to negations and 38 were due to other subject mention. The total precision was 80%. Looking at the two types seperately result in a 84% precision for the negations, and a rather low 45 % for the subject. Not all negations performed the same as evidenced by the breakdown below.

| Negation | Total occurences | Correctly negated | Precision |
| --- | --- | --- | --- |
| INGEN (no) | 196 | 183 | 93.4 % |
| IKKE (not) | 173 | 132 | 76.3 % |
| UDEN (without) | 48 | 33 | 68.8 % |
| ALDRIG (never) | 10 | 10 | 100 % |
| INTET (no) | 6 | 4 | 66.7 % |

The total occurences sum to 433, since some hits contained more than one negation.

In summrary, the textmining approach matches a total of 3135 terms, out of which 411 were disqualified due to negations. Of these disqualified hits 84 % (330 cases) were correctly disqualified. Of the 2,724 hits that were not disqualified 4.5% (122 cases) were missed by our negation module. That means our negation module identified 73% (330/(330+122)) of those negations that were relevant to the mined hits, and was correct in 84% of the detected cases. A more complicated negation detection system could possibly reduce the number of hits that are falsely thrown away and falsely included, but we find that our system ensures sufficient quality of the data that is used in the downstream analysis.

**Genetic overlaps between ICD10 pairs**

For the purpose of finding genetic overlaps between diseases present in the corpus, we used a previously generated and validated experimentally derived human protein interaction network [1,2,3]. The underlying data in this network stems from the databases BIND [4], MINT [5], IntAct [6], KEGG [7], Reactome [8], HPRD [9], DIP [10] and GRID [11]. Data is transferred between organisms using the Inparanoid orthology database [12] as previously described in the aforementioned publications. This protein-protein interaction network (PPI) contains 350,029 unique interactions between 12,507 human proteins. The first order interactors from OMIM disease causing genes (seed genes) in this network were extracted, after considering an optimal cutoff separating signal from noise in the PPI network [2], thus creating a disease network. Genetic overlap was detected between two diseases if they had shared genes that are first order interactors with the seed genes in the disease network.

**Bibliography**

1. Lage K, Møllgård K, Greenway S, Wakimoto H, Gorham JM, et al. (2010) Dissecting spatio-temporal protein networks driving human heart development and related disorders. Mol Syst Biol 6: 381.

2. Lage K, Hansen NT, Karlberg EO, Eklund AC, Roque FS, et al. (2008) A large-scale analysis of tissue-specific pathology and gene expression of human disease genes and complexes. Proc Natl Acad Sci USA 105: 20870-20875.

3. Lage K, Karlberg E, Størling Z, Ólason P (2007) A human phenome-interactome network of protein complexes implicated in genetic disorders. Nature Biotechnology.

4. Bader GD, Betel D, Hogue CWV (2003) BIND: the Biomolecular Interaction Network Database. Nucleic Acids Res 31: 248-250.

5. Zanzoni A, Montecchi-Palazzi L, Quondam M, Ausiello G, Helmer-Citterich M, et al. (2002) MINT: a molecular interaction database. FEBS Lett 513.

6. Hermjakob H, Montecchi-Palazzi L, Lewington C, Mudali S, Kerrien S, et al. (2004) IntAct: an open source molecular interaction database. Nucleic Acids Res 32.

7. Kanehisa M, Goto S (2000) KEGG: kyoto encyclopedia of genes and genomes. Nucleic Acids Res 28: 27-30.

8. Matthews L, Gopinath G, Gillespie M, Caudy M, Croft D, et al. (2009) Reactome knowledgebase of human biological pathways and processes. Nucleic Acids Res 37: D619-622.

9. Keshava Prasad TS, Goel R, Kandasamy K, Keerthikumar S, Kumar S, et al. (2009) Human Protein Reference Database--2009 update. Nucleic Acids Res 37: D767-772.

10. Salwinski L, Miller C, Smith A, Pettit F, Bowie J, et al. (2004) The database of interacting proteins: 2004 update. Nucleic Acids Res 32.

11. Breitkreutz B-J, Stark C, Reguly T, Boucher L, Breitkreutz A, et al. (2008) The BioGRID Interaction Database: 2008 update. Nucleic Acids Res 36: D637-640.

12. O'Brien K, Remm M, Sonnhammer E (2002) Inparanoid: a comprehensive database of eukaryotic orthologs. Nucleic Acids Res 33: D476.
